# The Medical Reporter

**Published:** 1853-08

**Authors:** 


*The Medical Reporter.* A Quarterly Journal; published under the direction of the Chester and Delaware County (Penn.) Medical Societies.

This new periodical starts under the auspices of the societies above named, with the intention of making it a medium for their transactions. This is a good idea, but we cannot subscribe to the Latin motto on its title page; even if Seneca did say it. "There is never too much said, because there is never enough learned."—(Trans.) But alas; much talking does not imply much learning. S. B. H.

